# A Case of Entire Obstruction of the Bowels for Three Weeks

**Published:** 1880-04

**Authors:** 


*A Case of Entire Obstruction of the Bowels for Three Weeks—Temporary relief afforded by Colotomy.*—The paper read by Dr. A. C. Post on the above subject being before the Academy for Discussion, Dr. Jas. L. Little referred to a case as follows: About four years ago he saw a patient in Vermont who had had complete intestinal obstruction for about sixty days. The patient had had difficulty in having passages from the bowels for some eight or ten years previously, and finally complete obstruction occurred. For two weeks, the patient had vomited more or less, had hiccup, the abdominal walls were distended, and the only relief which Dr. Little could suggest was in the operation of colotomy. No definite cause for the obstruction could be found by digital examination. He had a great variety of internal treatment, but entirely without benefit. Colotomy was performed, and an enormous quantity of fæcal matter, at least three quarts of semi-solid clay-colored material, came through the wound during the first twenty-four hours after the operation. The patient recovered, was still living, and yet had the artificial anus. One peculiarity of the case was the diffi culty in keeping the wound open. About two months after the operation the parts were united completely, and Dr. Porter, the attending physician, was obliged to make an incision, empty the colon, and afterward to maintain the tegumentary opening by means of tents. The opening however, closed the second time, and the patient went without any discharge of fæces for six weeks. The knife was again used, but the wound again closed entirely; and that was repeated in all four times, when it remained open permanently. No fæces had passed through the rectum. Before the operation a flexible tube could be easily pushed up into the bowel for the distance of two and a half feet, and through it injections were given, but without avail, although occasionally, when the tube was with drawn, its extremity was smeared with fæcal matter The only point at which there was anything that felt like a tumor was just within reach of the finger. The only diagnosis made was probable stricture high up in the colon.

Dr. Garrigues referred to a case of intestinal obstruction occurring in a nun æt. 20 years. No passage from the bowels occurred between August 4th and August 18th. She had vomited everything that had been put into the stomach, but no fæcal matter. A variety of remedies were used without benefit. A tube was introduced as high as the junction of the descending with the transverse portion of the colon, and injections given several times. The last one given contained a teaspoonful of green soap, and all at once the patient felt as though something had given way, and was at once relieved. Two days afteward a large pultaceous evacuation from the bowels occurred. He had at one time contemplated laparotomy for the relief of volvulus, and the sudden getting well suggested that it may have existed. He asked whether any of the members had used quicksilver in cases of intestinal obstruction—a remedy which at one time was recommended and used in doses of a pound or two.

Dr. Post remarked that he had a brother who had a scirrhus contraction of the rectum, and in his case quicksilver was used, and it passed through, but brought nothing with it. He lived twenty-nine days without a fæcal evacuation. At that time the present modes of relief were not usually practiced, and he was allowed to die with complete obstruction of the bowels.

The President alluded to cases of lead colic, in which quicksilver had been used in half pound doses. In one case the quicksilver passed through without the passage of any fæcal matter, was collected by the patient, placed in a bottle, and carried about as a trophy.

Dr. J. Marion Sims then read a paper on hydrobromic ether—bromide of ethyl—as an anæsthetic.

The paper being before the Academy, the discussion, on invitation by the President, was opened by Dr. Levis, of Philadelphia, who made some general remarks which were contained in a paper upon the subject to be published in the *Medical Record.* The only explanation he could give with reference to the difference in results obtained by Dr. Sims and himself was, that there might be a difference in the method in which it was used. He thought that some of the errors were such as he made in his first cases in which he administered it, when he began very cautiously. He now knew that the proper method of administration was to make a rapid impression, such as was done in giving nitrous oxide gas. He had been able, nearly always, to produce complete anæsthesia, by the use of two drachms, in two or three minutes. He had produced anæsthesia in forty-five seconds; but if the patient could be made to inspire and expire freely, the average time was about three minutes. The only point in Dr. Sims's case which impressed him especially was the duration of the operation, and the large quantity of the anæsthetic used. In his own cases the operation had not lasted longer than forty minutes, and the entire quantity of the ethyl used was eleven drachms. He had not seen any after effects whatever. As to nausea and vomiting, it perhaps was almost as likely to occur after the use of the ethyl as after the use of ether, if a considerable quantity was given. The quality of the article he regarded as very important, and he was sure that, since he had used Wyeth's preparation, there had been less tendency to nausea. With reference to the persistent odor in the breath, vomited matters, etc., he had noticed it when he had used the same preparation that Dr. Sims used.

The President—It will be noticed that the symptoms and phenomena manifested differ entirely from the symptoms and phenomena manifested after the administration of any other anæsthetic which has been used. I therefore take the privilege of calling upon Dr. Squibb, of Brooklyn, with the view of receiving an explanation of its failure to be eliminated from the system; that is, whether there are any constituents, either bad or good, in the ethyl, which of itself might destroy life.

Dr. E. R. Squibb—I fear, Mr. President, I have but little to add to the elucidation of this subject, and I must premise my remarks by saying that all I have to say upon the question is entirely theoretical. I confess to having had a prejudice against this agent from the earliest I knew anything of it, and it is upon theoretical principles, and upon those only, that I am capable of speaking or throwing out hints which possibly may be useful. As a chemical rule, I think that the anæsthetics which are the least dangerous are those which are the most simple, and when decomposed yield elements which, physiologically, are known to be most innocuous. If we commence with the nitrous oxide, we find that it is decomposable only into innocuous elements. If we go one step further and take the oxide of ethyl (and here I wish to protest against the bromide of ethyl being called an ethyl,) it can be decomposed into comparatively innocuous elements. In the bromide of ethyl the oxygen of the ordinary ether is replaced by bromine, and the compound contains 73 per cent. of bromine, the other 27 per cent. being the ethyl. Now, when we compare the effects of all the known anæsthetics, we will find that those are more easily borne which are simplest in their nature; that is to say, the compound ethers are less safe than simple ethers, and both the compound and the simple ethers seem to be proportionately safe to the simplicity and innocuousness of the elements of which they are composed.

We all know that bromine is an irritant poison. We all know that bromide of ethyl is a loosely molecular article, is decomposed very easily, much more so than bromides of ordinary bases which act as salts and are eliminated as such. Now we will suppose that the bromide of ethyl, under certain circumstances, is decomposed and its irritant property, 73 per cent. of bromine, goes into the system; it is easy to understand that grave effects might be produced, and perhaps we shall be able in this way to account for the exceptional cases in which the anæsthetic has been used with unpleasant results, whereas, if it had remained as bromide of ethyl, it may not have been harmful.

Let us take an analogy found in chloroform, which contains 89 per cent. of chlorine. Chlorine is one of the organic bodies which does not possess the irritant properties that bromine does, and two atoms of chlorine are not so active in producing toxic effects as one atom of bromine found in the bromide of ethyl. The point can be illustrated still further by supposing the existence of an arsenite of ethyl, the activity of which in producing dangerous effects as compared with the bromide of ethyl would be represented by the difference between the activity of bromine and the activity of arsenic as irritant poisons. If it is admitted that such theoretical argument has any value, I think we could understand some of the dangerous effects produced when compound ethers are used as anæsthetics; and I think Richardson's observations point us toward the conclusion that compound ethers are less safe still than the oxides. This brings us down to the oxides and nitrogenous oxides of ethyl, which are the safest of all known anæsthetics.

There is another point; and that is, any agent that can be powerful and active for good, can be powerful and active for evil. We see that in chloroform, and we see it in the bromide of ethyl. I think we can logically argue something from the chemical composition of agents as to which will be useful to us, and especially in the application of agents which carry us so near the dividing-line between life and death.

Dr. J. C. Dalton—I should be very happy, Mr. President, to give to the Academy, in response to your invitation, any information, did I have it, with regard to this subject. But unfortunately I am worse off than Dr. Squibb, for I had never even seen any hydrobromic ether before this evening. There were some points in Dr. Sims's paper, however, which struck me forcibly, and one is the different kinds of danger which may be developed by different kinds of anæsthetics. I suppose that the profession are tolerably well convinced now that ether, when it is dangerous as an anæsthetic, is mainly so in consequence of the presence of some morbid change in some vital organ; that when the entire body is perfectly healthy, ether comes as near to being a perfectly safe anæsthetic as we can have. I do not know of any modification with regard to that statement.

In this case, reported by Dr. Sims, if the death was due to the hydrobromic ether, it was a death which took place long after the substance was administered. It was a death apparently due to the persistent obstinate possession which the bromide of ethyl had taken of the entire system. The system could not get rid of the substance with which it was saturated, and which in some unexplained way produced those symptoms which have been so faithfully detailed. I do not wish to proffer any opinion regarding the cause of death in the case, but it seems that there was repetition of peculiar symptoms, in the midst of which the patient finally died, and that after death the entire system did retain, during a long period, the bromide of ethyl in sufficient quantity to indicate its active properties. How entirely different deaths from chloroform take place. They occur without any convulsions, without any marked symptoms, and odoriferous bodies of persons who have died under its influence have not been specially observed. I do not suppose, however, we have yet exhausted all the methods by which various anæsthetics are dangerous or may prove fatal. But the difference between the manner in which death is produced by chloroform and by ether when given to animals has, in my experience, been very striking; so much so that I long ago abandoned the habitual use of chloroform while experimenting, because it was so annoying to lose an animal just before the experiment was required and could not be postponed. Not long since, in order to avoid the disturbance produced by ether, I chloroformed a cat for the purpose of making an ophthalmoscopic examination. With all the recollection of frequent previous disappointment fresh in mind, I was extraordinarily careful in giving the anæsthetic, both with reference to quantity and time occupied in its administration and all the circumstances that could effect the result, and yet, just as I wished to use the instrument, the animal was dead. Here, however, we seem to have another mode of death, and one which, if real, is perhaps as difficult to avoid as sudden death in the use of chloroform. I am unable to see how any greater care could have been used than was exercised by Dr. Sims, although it is possible that Dr. Levis, who has a much greater experience in the use of the agent, might point out some improvement in the method adopted.

Dr. A. C. Post remarked, with reference to the prolonged action of anæsthetic agents, that he had been in the habit, for several years, in cases in which he anticipated a long operation, to use Prof. Nussbaum's method, premising the use of the anæsthetic by the hypodermic injection of seven or eight minims of Magendie's solution of morphia. He had found by doing so that the anæsthetic effect could be kept up for a long time with more quietude and less quantity of the anæsthetic than where the morphia had not been thus administered, and he was inclined to think it added to the safety of the patient as well as to the comfort of the surgeon.

Dr. Jas. L. Little referred to two cases, as follows: Case 1.—Last Tuesday, having received a specimen of the bromide of ethyl from Wyeth of Philadelphia, he determined to test its anæsthetic properties, and accordingly gave it to a boy three or four years old, upon whom he proposed to perform Heaton's operation for the radical cure of hernia. The ethyl was administered in the manner proposed by Dr. Levis; probably less than *two* drahms were used, and the patient went under its influence in less than two minutes. The pupils were contracted. Within five minutes after the cloth was removed from his face the child was able to respond intelligently. There was no vomiting.

Case II.—An adult, in good general condition, had two or three drachms held close over his mouth and nose on a napkin, and at the end of three minutes, no special effect had been produced by the anæsthetic except to excite powerful struggling on the part of the patient. The ethyl was applied more freely, the patient struggled very violently, the face was suffused, not however like that seen in ether inhalation; the pupils dilated and contracted alternately, the eyeballs rolled about; and at the end of *eight* minutes Dr. Little became somewhat alarmed at the condition of the man and discontinued the ethyl. The patient seemed, within three or minutes, to come from under what influence of the anæsthetic he had received. The quantity used was *two ounces* and *two drachms* by weight, and the time occupied *eight* minutes. There was neither nausea nor vomiting. Both the pulse and the respiration became exceedingly rapid. The condition was entirely different from anything he had ever seen in connection with the inhalation of either chloroform or ether. Ether was subsequently administered, and the operation performed.

The President—It will be noticed that Dr. Sims reported in his case that the respiration became exceedingly rapid. In the administration of this anæsthetic it seems, from what has been said, that it is necessary to produce its anæsthetic effect as rapidly as possible; that no atmospheric air is admitted, or just as little as possible. Of course, associated with this is another influence. We know that the exhalation of carbonic acid gas is limited, and, therefore, the question naturally arises: May not that fact be one which contributes to the danger; may not a part of the poisoning be due to the carbonic acid gas? I will next call upon Dr. Wylie, who has had experience with Dr. Sims in the administration of this agent.

Dr. Wylie remarked that he had nothing to add to the details of the case which Dr. Sims had reported, except anæsthetic that it had been used successfully. He thought it possible that in Dr. Sims's case sufficient stress had not been laid upon the operation itself as a factor in producing the death of the patient, as some deaths had followed the abdominal incision for removal of the ovaries, or Battey's operation. With reference to the tetanic convulsions, he thought they were not infrequently seen in connection with the use of ether. He also called to mind a case in which the patient had severe convulsions occurring with etherization, and it was subsequently found that the patient was an epileptic. It was possible that some such influence might have been at work in Dr. Sims's case. He thought that it might be difficult to use the ethyl in plastic operations about the face, because its effects were so evanescent.

The academy then adjourned.

